# Epicatechin Prevents Cryocapacitation of Bovine Spermatozoa through Antioxidant Activity and Stabilization of Transmembrane Ion Channels

**DOI:** 10.3390/ijms24032510

**Published:** 2023-01-28

**Authors:** Štefan Baňas, Filip Benko, Michal Ďuračka, Norbert Lukáč, Eva Tvrdá

**Affiliations:** 1 Institute of Biotechnology, Faculty of Biotechnology and Food Sciences, Slovak University of Agriculture in Nitra, Tr. A. Hlinku 2, 949 76 Nitra, Slovakia; 2 Institute of Applied Biology, Faculty of Biotechnology and Food Sciences, Slovak University of Agriculture in Nitra, Tr. A. Hlinku 2, 949 76 Nitra, Slovakia; 3 AgroBioTech Research Centre, Slovak University of Agriculture in Nitra, Tr. A. Hlinku 2, 949 76 Nitra, Slovakia

**Keywords:** epicatechin, cryopreservation, spermatozoa, bulls, sperm vitality, oxidative stress, cryocapacitation, protein expression

## Abstract

Epicatechin (EPC) is a flavonoid belonging to the family of catechins; it has been described as a powerful scavenger of a wide spectrum of reactive oxygen species (ROS) and a modulator of ex vivo sperm vitality. In this study, we assessed the potential protective abilities of EPC on cryopreserved bovine spermatozoa. We focused on conventional quality parameters, as well as the oxidative profile of spermatozoa alongside capacitation patterns, and expression profiles of proteins involved in the process of capacitation. Semen samples were cryopreserved in the presence of 25, 50 or 100 μmol/L EPC and compared to native semen (negative control) as well as ejaculates frozen in the absence of EPC (positive control). A dose-dependent improvement of conventional sperm quality parameters was observed following EPC administration, particularly in case of the sperm motility, membrane, acrosome and DNA integrity in comparison to the positive control. Experimental groups exposed to all EPC doses presented with a significantly lower proportion of capacitated spermatozoa as opposed to the positive control. While no significant effects of EPC were observed in cases of superoxide production, a significant decrease in the levels of hydrogen peroxide and hydroxyl radical were recorded particularly in the experimental groups supplemented with 50 and 100 μmol/L EPC. Western blot analysis revealed that supplementation of particularly 100 μmol/L EPC to the semen extender prevented the loss of the cation channel of sperm (CatSper) isoforms 1 and 2, sodium bicarbonate cotransporter (NBC) and protein kinase A (PKA), which play important roles in the process of sperm capacitation. In summary, we may hypothesize that EPC is particularly effective in the stabilization of the sperm membrane during the freeze–thaw process through its ability to quench ROS involved in damage to the membrane lipids and to prevent the loss of membrane channels crucial to initiate the process of sperm capacitation. These attributes of EPC provide an additional layer of protection to spermatozoa exposed to low temperatures, which may be translated into a higher post-thaw structural integrity and functional activity of male gametes.

## 1. Introduction

Cryopreservation enables long-term storage of spermatozoa in a state of metabolic arrest that prevents their senescence while maintaining their structural integrity, vitality and fertilizing potential, thus enabling their use when and where needed [1]. This technique is particularly valuable in animal production, since it allows a compelling utilization of genetically valuable stud males with high reproductive efficiency, leading to desirable fertility rates and litter size, while reducing the risks of horizontal or vertical transmission of venereal diseases among animals [2,3]. At the same time, implementation of sperm cryopreservation followed by artificial insemination (AI) cuts down production costs by decreasing the number of stud males, and thus, saving space, feed, maintenance and operating expenses [2,4].

A decisive factor in sperm cryobiology lies in the understanding that male gametes are small cells with a relatively large surface [5] that will have an impact on the viscosity and transition temperature of their intracellular environment [1]. If cryoprotective agents are absent during the cryopreservation procedure, thermal shock, osmotic stress and ice crystals may contribute to irreversible alterations to the structures critical for sperm survival [1,6]. Oxidative stress, caused by reactive oxygen species (ROS) overproduction during cryopreservation, is considered a hallmark of impaired sperm quality following the freezing and thawing process [7]. This is primarily caused by the presence of high amounts of polyunsaturated fatty acids (PUFAs) in the sperm plasma membrane and their susceptibility to interact with ROS and to subsequently disintegrate into peroxyl radicals and lipid hydroperoxides [8]. In addition, the seminal plasma as an important antioxidant reservoir is diluted with the semen extender and, hence, is insufficient to cope with higher ROS levels released during cryopreservation [9]. As such, lipid peroxidation [10,11], protein carbonylation [11,12] and accumulation of DNA adducts [12,13] have been often observed in cryopreserved spermatozoa.

Currently, premature capacitation ought to be a central side effect of sperm cryopreservation. As opposed to physiological capacitation, cryocapacitation is triggered by temperature fluctuations, ice crystals and oxidative insults, leading to the loss of transmembrane channels responsible for the transport of calcium (Ca^2+^) and bicarbonate (HCO_3_^−^) crucial for the initiation of capacitation [14]. Besides a decreased production of cyclic adenosine monophosphate (cAMP) necessary for the initiation of hyperactivated sperm motility [15], low temperatures may alter the sperm membrane fluidity, which will be accompanied by the loss of proteins, cholesterol and phospholipids; this results in compromised cells that interact with the chlortetracycline hydrochloride (CTC) stain traditionally used to detect and/or monitor physiological capacitation [14,16]. From a functional point of view, if frozen spermatozoa capacitate immediately following thawing, these will quicky consume energy otherwise necessary to reach and fertilize the ovum [17], rendering the semen sample as unsuitable for AI. As such, substantial effort is currently being invested into new strategies to prevent sperm deterioration during or following the freeze–thaw process.

For several decades now, different attempts have been made to reduce the detrimental effects of cryopreservation on spermatozoa. The current approach is based on so-called defensive strategies, in which different supplements are added to freezing media to offer protection against cryodamage to male gametes. Cryoprotective agents [18], antioxidants [19], antifreeze proteins [20], fatty acids [21] or energetic molecules [22] are amongst the most studied. Nevertheless, current attention has increasingly shifted to natural biomolecules and extracts derived from medicinal herbs used as remedies in traditional medicine with a variety of beneficial effects on male reproduction [23,24,25].

Catechins are polyphenolic flavonols, which are the primary bioactive molecules of green tea. Besides, strawberries, black grapes, cocoa and apricots are also reported to be rich in catechins [26]. The family of green tea catechins comprises four different biomolecules, specifically epicatechin (EPC), epicatechin-3-gallate, epigallocatechin and epigallocatechin-3-gallate [27], all of which have been studied as nutraceuticals in the prevention of malignancies, cardiovascular, metabolic or neurological diseases [26,28]. Epicatechin is most notably known as a strong ROS-scavenger [29] and chelator of heavy metals, such as chromium and cadmium [30]. At the same time, the molecule is able to reduce iron and copper and thus prevent the Fenton reaction involved in the production of hydroxyl radical, considered to be the most toxic and aggressive reactive intermediate [27,29].

Over the past years, it has been suggested that catechins are able to modulate the levels of monoamines, which are involved in sexual and reproductive behavior, leading to enhanced sexual activity [31]. Subsequent in vivo studies have revealed that catechin administration ameliorated testicular damage caused by radiation [32], heat stress [33], chemotherapy [34] or environmental pollutants [35]. Moreover, in vitro reports have indicated potential benefits of catechins as supplements to extenders for buck [36], boar [37], bull [11] and dog [38] semen. Most of these agree that catechins, and specifically EPC, may provide additional ex vivo protection against oxidative damage to the membranous structures of male gametes, including the plasma membrane and mitochondria [11,39,40]. Recent research from our group has also revealed that EPC could be effective in preventing the loss of heat shock proteins 70 and 90 during the freeze–thaw process of bovine spermatozoa [41]. Furthermore, it has been hypothesized that EPC could interfere with the process of sperm capacitation and acrosome reaction by modulating cholesterol efflux from the membranes [42]. Nevertheless, the mechanisms interconnecting the ability of EPC to act as an antioxidant on one hand and a sperm capacitation modulator on the other are still poorly understood.

As such, in this study, we strived to assess the impact of epicatechin on fluctuations in the most notorious ROS involved in sperm cryodamage in a broader context of changes to quality characteristics of cryopreserved bovine spermatozoa. At the same time, we studied the potential of epicatechin to prevent cryocapacitation through expression patterns of the cation channels of sperm (CatSper) isoforms 1 and 2 (CatSper1 and CatSper2), sodium bicarbonate cotransporter (NBC) and protein kinase A (PKA), considered to play elementary roles in the process of capacitation.

## 2. Results

### 2.1. Conventional Sperm Quality Parameters

**Figure 1:**
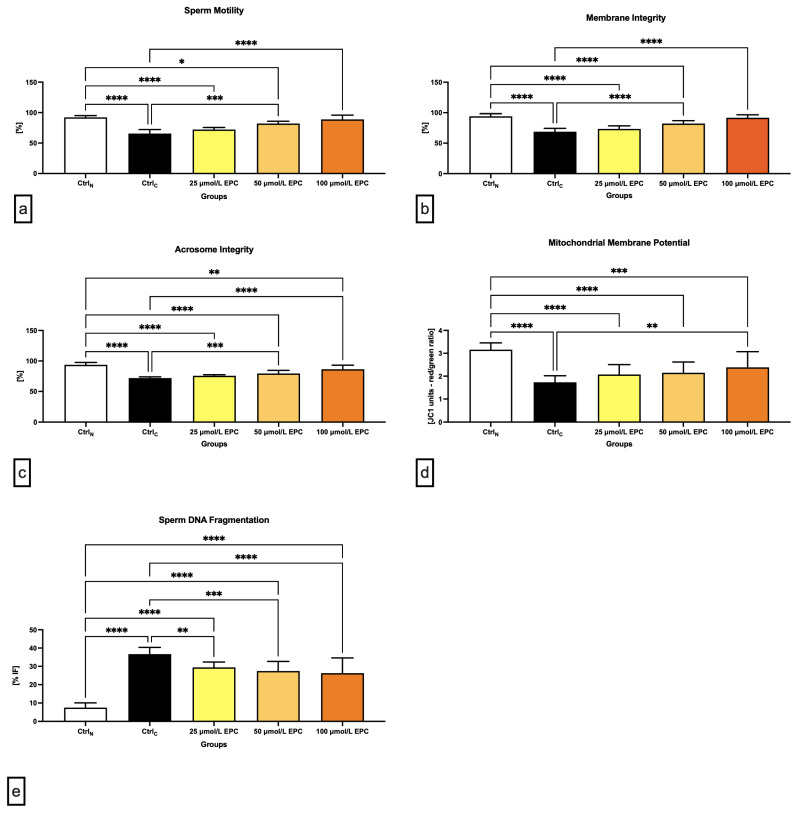
Conventional semen quality parameters including (**a**) motility [%]; (**b**) membrane integrity [%]; (**c**) acrosome integrity [%]; (**d**) mitochondrial membrane potential [red/green ratio]; and (**e**) DNA fragmentation [% index of fragmentation] of bovine spermatozoa cryopreserved in the presence of three doses of epicatechin. Mean ± S.D. CtrlN—native spermatozoa (negative control); CtrlC—spermatozoa cryopreserved in the absence of epicatechin (positive control). **** *p* < 0.0001, *** *p* < 0.001, ** *p* < 0.01, * *p* < 0.05.

Data obtained from the computer-aided sperm analysis (CASA) revealed a significant (*p* < 0.0001) decrease of the sperm motility of the cryopreserved control group (CtrlC; Figure 1a) following thawing. Exposure of spermatozoa to 100 μmol/L EPC led to a significant improvement of the motion behavior (*p* < 0.0001) when compared to CtrlC. Significant differences in the sperm motility were also detected between the native control (CtrlN) and experimental groups exposed to 25 μmol/L EPC (*p* < 0.0001) and 50 μmol/L EPC (*p* < 0.05). Generally speaking, the higher the EPC concentration, the better protection was delivered to the sperm motion activity of cryopreserved spermatozoa.

Correspondingly to an improvement of post-thaw motility, a significantly increased membrane integrity was detected in experimental groups exposed to 50 μmol/L EPC and 100 μmol/L EPC in contrast to CtrlC (*p* < 0.0001; Figure 1b). In comparison with the native control group, significant differences were observed in case of experimental groups supplemented with 25 μmol/L EPC and 50 μmol/L EPC (*p* < 0.0001).

Thermal shock also exhibited negative effects on the sperm acrosome integrity. When compared to CtrlN, a significant decrease of the integrity of acrosome was recorded in CtrlC as well as in experimental groups supplemented with 25 μmol/L EPC and 50 μmol/L EPC (*p* < 0.0001; Figure 1c). Inversely, a significant improvement of the acrosome stability in comparison to CtrlC was observed following the administration of 100 μmol/L EPC (*p* < 0.0001).

Detrimental effects of sperm cryopreservation on the mitochondrial activity were unraveled by the JC-1 assay. Significant differences in the mitochondrial membrane potential were recorded among CtrlN and CtrlC (*p* < 0.0001; Figure 1d) as well as CtrlN and all EPC-supplemented experimental groups (*p* < 0.0001 in case of 25 μmol/L EPC and 50 μmol/L EPC; *p* < 0.001 with respect to 100 μmol/L EPC), suggesting that none of the EPC doses was effective enough to prevent cryodamage to sperm mitochondria. Nevertheless, a significant improvement of the mitochondrial membrane potential was observed in the experimental group administered with 100 μmol/L EPC (*p* < 0.01) as opposed to CtrlC.

Increased occurrence of spermatozoa with structural and functional alterations following the freeze–thaw process was accompanied by a rise of sperm DNA damage, as observed by significant differences in the index of DNA fragmentation among the native and cryopreserved control (*p* < 0.0001; Figure 1e). The comparative analysis also revealed a significant increase of DNA damage in spermatozoa exposed to all EPC doses (*p* < 0.0001). Nevertheless, the fragmentation index was significantly lower following the administration of 50 μmol/L EPC and 100 μmol/L EPC in comparison to the untreated cryopreserved control (*p* < 0.001 with respect to 50 μmol/L EPC; *p* < 0.0001 in relation to 100 μmol/L EPC).

### 2.2. Capacitation Patterns

**Figure 2:**
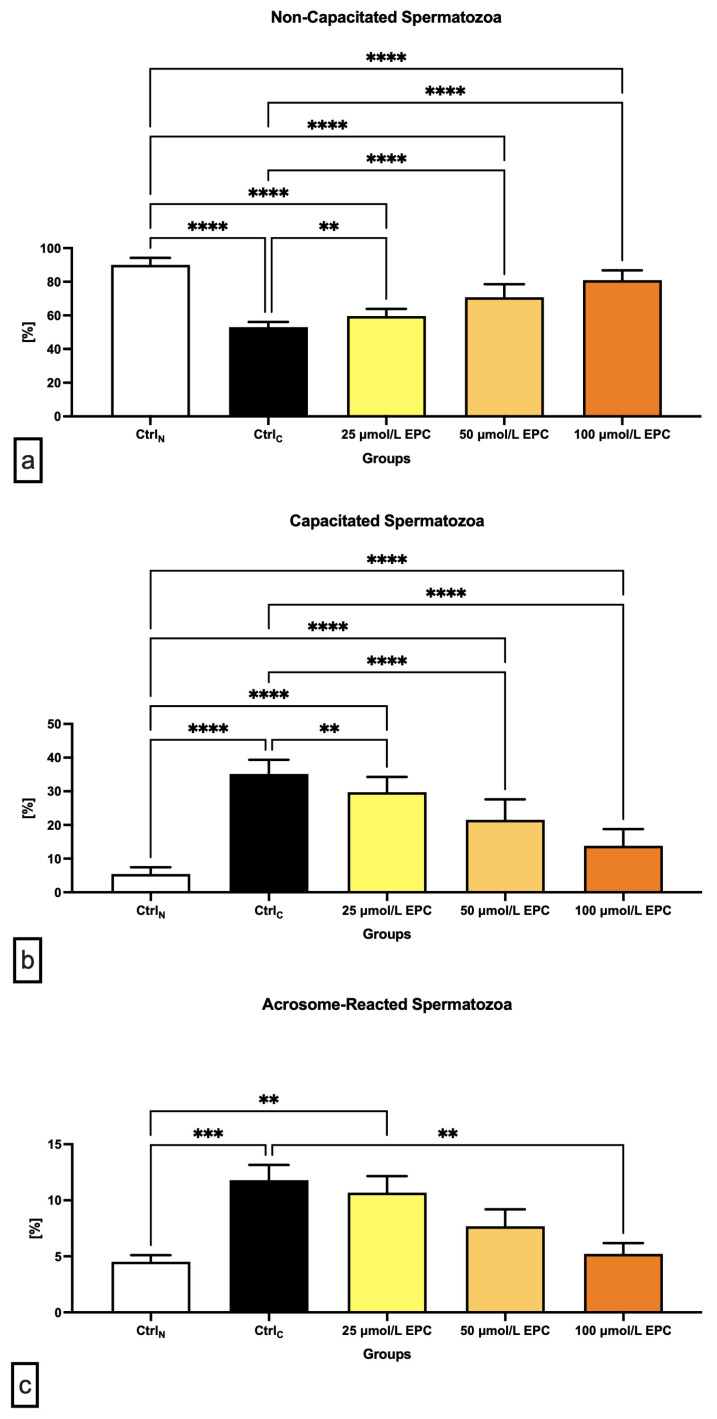
Proportion of (**a**) non-capacitated; (**b**) capacitated; and (**c**) acrosome-reacted [%] bovine spermatozoa cryopreserved in the presence of three doses of epicatechin. Mean ± S.D. CtrlN—native spermatozoa (negative control); CtrlC—spermatozoa cryopreserved in the absence of epicatechin (positive control). **** *p* < 0.0001, *** *p* < 0.001, ** *p* < 0.01.

The lowest proportion of non-capacitated spermatozoa (“F”-pattern) was recorded in the cryopreserved control, which was significantly different in comparison with the native control (*p* < 0.0001; Figure 2a). A significant decrease of non-capacitated spermatozoa was also observed in all experimental groups containing EPC in contrast to CtrlN (*p* < 0.0001). On the other hand, supplementation of all EPC doses led to at least partial prevention of the loss of non-capacitated spermatozoa when compared to the control group cryopreserved in the absence of EPC (*p* < 0.01 with respect to 25 μmol/L EPC; *p* < 0.0001 in case of 50 μmol/L EPC and 100 μmol/L EPC).

Parallel to the above-mentioned data, a significant increase of “B”-pattern spermatozoa, consistent with the onset of capacitation, was observed in the cryopreserved control in comparison to the native control (*p* < 0.0001; Figure 2b). EPC was not able to fully prevent premature capacitation as observed by a significantly higher proportion of B-pattern spermatozoa in all experimental groups in comparison to CtrN (*p* < 0.0001). Nevertheless, the presence of capacitated spermatozoa in the experimental groups was significantly lower when compared to CtrlC (*p* < 0.01 with respect to 25 μmol/L EPC; *p* < 0.0001 in relation to 50 μmol/L EPC and 100 μmol/L EPC).

Along with changes to the acrosome integrity observed in the pre-established control and experimental groups, a significant increase of “AR”-pattern spermatozoa, indicating a premature acrosome reaction, was observed in the CtrlC group as opposed to the native control (*p* < 0.001; Figure 2c). On the other hand, administration of 100 μmol/L EPC to the semen extender resulted in a significantly decreased occurrence of acrosome-reacted spermatozoa in comparison to the cryopreserved control (*p* < 0.01).

### 2.3. Oxidative Profile

**Figure 3:**
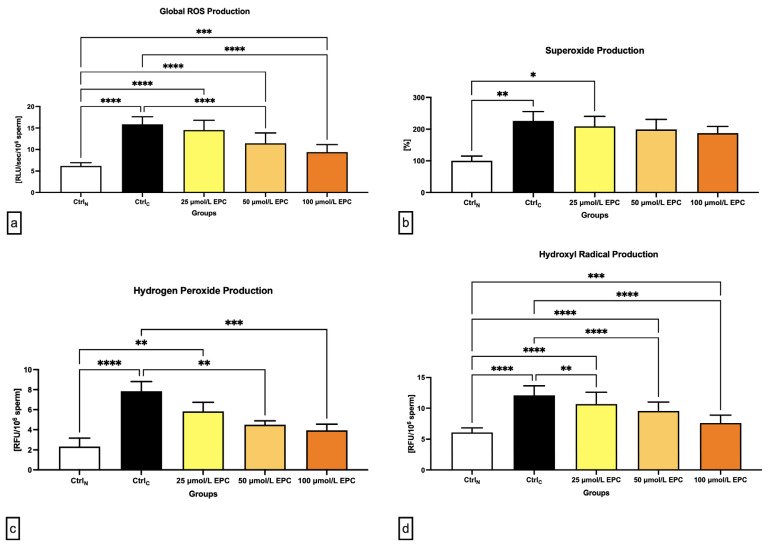
Oxidative profile including (**a**) global ROS production [RLU/sec/10^6^ sperm]; (**b**) superoxide production [%]; (**c**) hydrogen peroxide production [RFU/10^6^ sperm]; and (**d**) hydroxyl radical production [RFU/10^6^ sperm] by bovine spermatozoa cryopreserved in the presence of three doses of epicatechin. Mean ± S.D. CtrlN—native spermatozoa (negative control); CtrlC—spermatozoa cryopreserved in the absence of epicatechin (positive control). **** *p* < 0.0001, *** *p* < 0.001, ** *p* < 0.01, * *p* < 0.05.

As revealed in Figure 3a, global reactive oxygens species (ROS) production was significantly increased in all cryopreserved groups when compared to the native control (*p* < 0.0001 in case of CtrlC, 25 μmol/L EPC and 50 μmol/L EPC; *p* < 0.001 with respect to 100 μmol/L EPC), with the cryopreserved control containing the highest levels of reactive intermediates. Nevertheless, the comparative analysis revealed significantly lower ROS levels in experimental groups cryopreserved in the presence of 50 μmol/L EPC and 100 μmol/L EPC (*p* < 0.0001) as opposed to CtrlC.

In case of the superoxide (O_2_^-•^) production, a significant rise of its levels was observed in the CtrlC group and the experimental groups containing 25 μmol/L EPC in comparison to the native control (*p* < 0.01; Figure 3b). No significant differences among the experimental groups and the cryopreserved control were detected although a dose-dependent decrease of O_2_^-•^ concentrations was observed following EPC administration.

Similarly to O_2_^-•^, the highest levels of hydrogen peroxide (H_2_O_2_) were found in the cryopreserved control as opposed to the native control (*p* < 0.0001; Figure 3c). Significantly higher H_2_O_2_ concentrations in comparison to CtrlN were recorded in samples cryopreserved in the presence of 25 μmol/L EPC (*p* < 0.01). Inversely, it was revealed that administration of 50 μmol/L EPC and 100 μmol/L EPC to the semen extender led to a significant decrease of H_2_O_2_ production in contrast to the untreated cryopreserved control (*p* < 0.01 in case of 50 μmol/L EPC; *p* < 0.001 with respect to 100 μmol/L EPC).

Finally, a significant rise of hydroxyl radical (^•^OH) levels were noted in all pre-established groups subjected to the freeze–thaw process when compared to the native control (*p* < 0.0001 in relation to CtrlC, 25 μmol/L EPC and 50 μmol/L EPC; *p* < 0.001 with respect to 100 μmol/L EPC). These fluctuations mirrored changes observed in the quantification of global ROS levels, which makes us speculate that, in this study, ^•^OH accounted for the majority of ROS detected by the chemiluminescent assay. While EPC was not able to fully prevent the rise of ^•^OH levels in the experimental groups, a significantly lower ^•^OH production was observed in all groups cryopreserved in the presence of EPC (*p* < 0.01 with respect to 25 μmol/L EPC; *p* < 0.0001 with respect to 50 μmol/L EPC and 100 μmol/L EPC) when compared to the untreated cryopreserved control.

### 2.4. Western Blots

**Figure 4:**
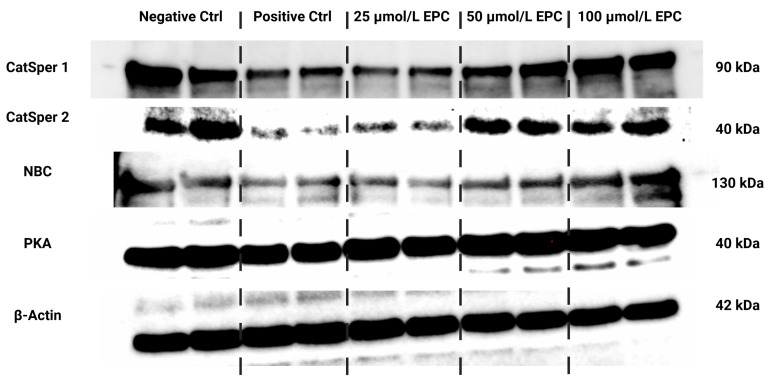
Protein expression patterns of CatSper1, CatSper2, NBC, PKA and β-Actin in bovine spermatozoa cryopreserved in the presence of three doses of epicatechin, as determined by Western blotting. Original photos of the gels, membranes and blots are available as Supplementary Materials. Created with (Supplementary: Confirmation of Publication and Licensing Rights) BioRender.com (accessed on 18 January 2023).

**Figure 5:**
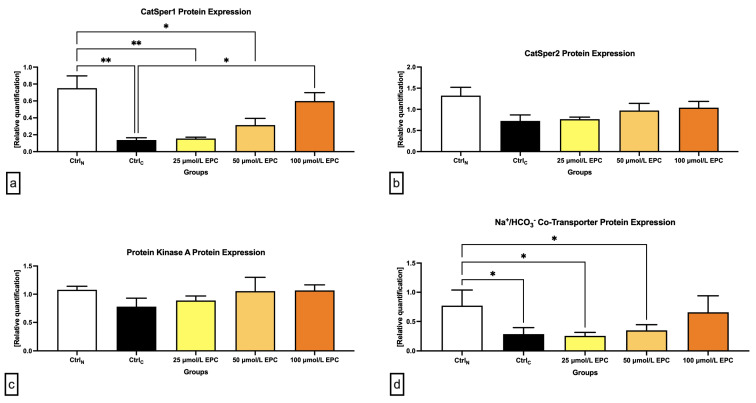
Graphical representation of the relative quantification of the (**a**) CatSper1; (**b**) CatSper2; (**c**) PKA; and (**d**) NBC protein in bovine spermatozoa cryopreserved in the presence of three doses of epicatechin. Mean ± S.D. CtrlN—native spermatozoa (negative control); CtrlC—spermatozoa cryopreserved in the absence of epicatechin (positive control). ** *p* < 0.01, * *p* < 0.05.

Data collected from the Western blot analysis revealed that CatSper1 expression in spermatozoa was significantly, negatively affected by the cryopreservation process in comparison to their native state (*p* < 0.01; Figure 4 and Figure 5a). In the meantime, all EPC doses supplemented to the cryopreservation medium provided a certain degree of protection to the protein following the freeze–thaw process. In particular, CatSper1 protein expression was significantly enhanced in the experimental group containing 100 μmol/L EPC (*p* < 0.001) when compared to the cryopreserved control.

No significant changes in the CatSper2 (Figure 4 and Figure 5b) and PKA (Figure 4 and Figure 5c) protein expression were detected when comparing the pre-established groups among each other although both proteins were notably underexpressed in the CtrlC group. A dose-dependent increase of the expression levels of both proteins was recorded in the experimental groups supplemented with EPC.

As revealed by Figure 4 and Figure 5d, the expression of the NBC protein was significantly reduced following the cryopreservation process as revealed by significant differences in its levels among the cryopreserved control and the native control (CtrlN; *p* < 0.05). A significant reduction of the protein in comparison to CtrlN was also observed in the experimental groups containing 25 μmol/L EPC and 50 μmol/L EPC (*p* < 0.05). Nevertheless, all EPC doses administered to the cryopreservation medium were able to at least partially prevent the loss of the NBC protein although no significant differences were observed.

## 3. Discussion

Sperm cryopreservation followed by AI are considered to be essential pillars of modern cattle production, and important contributors to the quality of animal products. Although cryopreservation of bovine spermatozoa is technologically advanced and well-managed, there are still major weaknesses present prior to and during the freeze–thaw process that hinder the use of its fullest potential in the breeding practice [43]. Since spermatozoa have lower water content and higher membrane fluidity, they should be in theory less sensitive to low temperatures in comparison to somatic cells. Nevertheless, a considerable portion of spermatozoa is lost during the cryopreservation process, rendering the specimen unsuitable for AI [1,3].

The principal sperm structure that is mostly affected by cryodamage is the plasma membrane, which will become more rigid and fragile; the phospholipid phase will be separated; and transmembrane proteins clustered irreversibly [44]. Such plasma membrane will lose its semi-permeable properties and integrity as observed in our study as well as in previous reports [17,45,46,47]. At the same time, cryopreservation has been associated with changes in the mitochondrial membrane fluidity that will, on one hand, result in ATP depletion, while damaged or dead mitochondria will release increased ROS amounts into the cell as observed in this study as well as in previous reports on frozen–thawed bovine spermatozoa [17,48,49]. Sperm lipids are notoriously known to be highly susceptible to oxidative damage due to exceptionally high amounts of polyunsaturated fatty acids [44]. Lipid peroxidation may in turn contribute to the disintegration of the membrane and acrosomal architecture, followed by an increased occurrence of toxic aldehydes, alkoxyl and peroxyl radicals, as well as lipid hydroperoxides [11,43,47]. As hypothesized by Benko et al. [17,50], axonemal proteins critical for the sperm movement, as well as mitochondrial enzymes responsible for ATP synthesis, may equally undergo oxidation, which leads to energy depletion and the loss of sperm motility. Besides, ROS overflow from peroxidized membranes and disintegrated mitochondria may trigger cell death [51].

Cryoshock-induced alterations to the key sperm structures may result in changes mimicking natural capacitation while severely damaging the cells which will become less stable upon their transfer to the female reproductive tract and with a significantly limited potential to reach and fertilize the oocyte. Our results have revealed that exposure of spermatozoa to low temperatures deregulates the expression of all studied transmembrane channels responsible for the transport of HCO_3_^−^ and Ca^2+^, which should subsequently stimulate cAMP synthesis and trigger the activity of PKA and tyrosine phosphorylases. This is in agreement with our previous study [14] as well as with reports on human [52] and rat [53] germ cells. The reasons for this under expression may be variable and are still subject to speculation. Flores et al. [54] suggests that this phenomenon be linked to alterations in mRNA–protein interactions and higher vulnerability of mRNA to degradation due to low temperatures. On the other hand, it has been hypothesized that transmembrane proteins could be leaking from already damaged membrane into the extracellular environment [55]. Finally, the loss of transmembrane channels could be linked to a higher occurrence of already dead cells unable to synthesize any protein anymore [3]. At the same time, we observed that PKA expression was reduced in untreated cryopreserved samples, which may provide a partial explanation to the loss of sperm motility and membrane integrity in the cryopreserved control, similarly to earlier studies on bovine [14] and fish [56] spermatozoa. Besides alterations in the downstream cascade, which is initiated by the activity of CatSper and NBC channels that stimulate PKA, the decline of the enzyme may have also been caused by the loss of A-kinase anchoring proteins which bind PKA to target proteins, as previously reported by Feliciello et al. [57]. This dysregulation may also be associated with the loss of acrosome integrity in the cryopreserved control since it has been reported that disruptions in the PKA anchoring process may lead to alterations in PKA localization within male gametes, leading to premature acrosome exocytosis [58].

Prevention or attenuation of oxidative damage to the sperm structures through supplementation of substances with the ability to oppose ROS generation or counteract oxygen-derived toxicity, has been achieved to a certain extent. Over the past years, numerous studies have emerged, suggesting that biomolecules from natural resources could improve the quality of stored semen more effectively than traditionally used antioxidants, such as vitamin C or vitamin E reviewed by [25,59,60,61]. In our case, the experimental design was built upon available evidence on the biological effects of EPC on male gametes, with the aim to shed more light on the mechanisms by which EPC exerts its protective and preventative properties against sperm deterioration inflicted by low temperatures.

The family of green tea catechins is known to comprise highly effective ROS-scavengers and metal chelators. These properties may be primarily attributed to the chemistry of the catechin polyphenols [62]. The basic structure of catechins consists of an ortho-benzoyl benzopyran derivative, which has more hydroxyl bases, and thus it easily interacts with an unpaired oxygen atom [63]. Once conjugated with oxygen, the benzene ring present in the catechin structure causes the hydrogen–oxygen bond to weaken, directly competing for active oxygen with unsaturated fatty acids, thus preventing their oxidation on one hand. On the other hand, once active oxygen and catechins bind, a stable dimer is formed, terminating the oxidation reaction [64]. This unique behavior enables catechins to act as exceptional antioxidants with a ROS-quenching power 20 times higher than that of vitamin C [65]. At the same time, nuclear magnetic resonance spectroscopy studies have unraveled the high affinity of catechins for the membranous structures of male gametes, primarily the plasma and mitochondrial membranes [66]. The high affinity for lipids, coupled with an inherent ability to bind to and dispose of reactive intermediates, provides explanation to a significant stabilization of the membrane integrity along with the maintenance of natural fluidity as uncovered by the CTC stain in this study, which may be considered as the primary mechanism of action by which EPC stabilizes the post-thaw vitality of spermatozoa. Accordingly, proper protection of mitochondrial structures, with a parallel prevention of ROS overload, may result in a higher proportion of spermatozoa with an active mitochondrial metabolism and desirable motility rates as previously observed in bulls [11,67], boars [37] and bucks [68].

Assessment of the ability of EPC to dispose of specific ROS classes supports the above-postulated hypothesis since the best efficiency of the polyphenol was observed in the case of H_2_O_2_ and ^•^OH. It is well-known that both H_2_O_2_ as well as ^•^OH possess high affinity to lipids and transfuse relatively easily through the membranes [69,70]. Once bound to the membranes, both ROS may initiate lipid peroxidation that will ultimately lead to the production of toxic metabolites such as malondialdehyde (MDA) and 4-hydroxynonenal considered as second messengers of oxidative stress [71]. What is more, both reactive intermediates may trespass the nuclear membrane and cause direct oxidative DNA degradation and cell death [72]. Since EPC is able to sharply capture ROS produced by the Fenton and Haber Weiss feedback [73] as well as peroxyl radicals, thus terminating the lipid peroxidation chain [62], we may speculate that this molecule may have a higher preference towards H_2_O_2_ and ^•^OH rather than O_2_^-•^ produced by the mitochondria. This assumption could be supported by earlier studies reporting on significantly lower levels of MDA, protein carbonyls and oxidative DNA adducts in bull [11,41,74], dog [38] and boar [75] spermatozoa preserved in the presence of catechin polyphenols or green tea extract. Furthermore, our hypothesis that the cytoplasmic membrane could act as a prime site of EPC activity may be further sustained by our earlier study revealing that the presence of particularly 50 μmol/L and 100 μmol/L EPC during bovine sperm cryopreservation led to a stronger protection of heat shock proteins located on the sperm surface [76] in comparison to the BAX and Bcl-2 proteins that are primarily mitochondria-bound [77].

The high ability of catechins to be incorporated into the plasma membrane was furthermore supported by our western blot analysis, indicating a stabilization of the CatSper and NBC transmembrane channels in the presence of particularly higher doses of EPC. This may be a consequence of the efficiency of EPC to dispose of ROS before these can reach and interfere with membrane lipids and thus stabilize the integrity and fluidity of the structural and functional components of sperm membranes. Besides, the ability of catechins to modulate the behavior of membrane-bound channels has been shown by De Amicis et al. [42], who reported that these polyphenols could avoid possible fluctuations in ion concentrations and alterations to Na^+^/K^+^ ATPase, which could have lethal consequences to the sperm survival. Furthermore, it was revealed that lower doses of catechins may modulate the cholesterol dynamics in the sperm membrane as well as tyrosine phosphorylation, which affects the sperm motility, viability and downstream phosphorylation protein and tyrosine-protein kinases. Correspondingly to the assessment of sperm capacitation patterns, we may assume that the presence of EPC during the freeze–thaw process protects the functional activity of membrane channels involved in a proper initiation and progress of capacitation by preventing lipid peroxidation that could endanger the stability of transmembrane ion carriers. At the same time, the cytoplasmic membrane, supported by an additional layer of antioxidant protection, will retain its integrity and semipermeable properties that will impede changes in its composition leading to premature capacitation and CTC-positivity. While the impact of EPC on the expression patterns of PKA was insignificant, a clear dose-dependent improvement of PKA expression was observed following the freeze–thaw process. This may be explained by the upstream stabilization of the transmembrane channels as discussed earlier since their proper activity leads to the stimulation of adenylyl cyclase and cAMP synthesis that will directly stimulate PKA. At the same time, the enzyme may be protected through a direct ROS-quenching ability of EPC that will dispose of ROS before they may reach the enzymatic machinery of the capacitation process that is located in the sperm head and neck [14]. This hypothesis is furthermore supported by an earlier report observing that supplementation of catechins to the semen extender stimulated adenylate cyclase/cAMP/PKA signaling in human sperm [42]. These findings were later corroborated by Spinaci et al. [78], according to whom epigallocatechin-3-gallate treatment increased the proportion of boar spermatozoa able to actively bind to the zona pellucida of the oocyte. Hence, it may be assumed that catechins could be able to modulate the process of capacitation, hyperactivation and acrosome reaction, even under ex vivo conditions.

Whilst the data collected from our experiments are encouraging, we must take several aspects into consideration in order to draw definitive conclusions. Since the capacitation machinery relies on a vast array of molecules, it is necessary to study the impact of EPC on further transmembrane channels responsible for the transport of ions through the membrane as well as downstream enzymes in charge of the acquisition of hyperactivated sperm motility and dynamic changes to the membrane architecture as a result of capacitation. Most importantly, the effect of EPC on the fertilization ability of bovine spermatozoa needs to be verified, either through in vitro fertilization experiments or more preferably via artificial insemination in the field.

## 4. Materials and Methods

### 4.1. Semen Collection and Cryopreservation

Semen samples were collected from 20 healthy and sexually mature Holstein–Friesian bulls (Slovak Biological Services, a.s., Nitra) on a regular collection schedule, with the help of an artificial vagina, and were subsequently transported to the laboratory in a Mini Bio Isotherm system (M&G Int, Renate, Italy).

Once in the laboratory, each ejaculate was divided into five equal aliquots. The first aliquot defined as the negative (native) control was diluted in Dulbecco’s phosphate-buffered saline (DPBS) (without calcium and magnesium; Sigma-Aldrich, St. Louis, MO, USA) to a final concentration of 44 million sperm/mL; it was then immediately evaluated. The remaining aliquots were processed with a semen extender comprising Triladyl (Minitub GmbH, Tiefenbach, Germany), 20% (*w*/*v*) fresh egg yolk, Tris, citrate, carbohydrates, buffering agents, glycerol, antibiotics and distilled water. In case of the positive (cryopreserved) control, the extender was supplemented with 0.5% DMSO (dimethyl sulfoxide; Sigma-Aldrich, St. Louis, USA). In the meantime, the extender for the experimental groups was supplemented with 25 μmol/L, 50 μmol/L or 100 μmol/L EPC (Sigma-Aldrich, St. Louis, MO, USA), previously dissolved in DMSO. Diluted semen samples were loaded into 0.25 mL French straws at 11 million sperm/straw and cryopreserved using a digital freezing machine (Digitcool 5300 ZB 250; IMV, L’Aigle, France), as previously described by Benko et al. [14]. The straws were kept in liquid nitrogen (−196 °C) for 6 weeks. Prior to the analyses, the cryopreserved samples were thawed in a water bath (37 °C) for 90 s, washed 3 times with DPBS and centrifuged at 300× *g* for 10 min to dispose of the egg yolk residues.

### 4.2. Conventional Sperm Quality Parameters

Sperm motion was assessed using computer-aided sperm analysis (CASA; Version 14.0 TOX IVOS II.; Hamilton-Thorne Biosciences, Beverly, MA, USA). Ten microliters of each sample were placed into a Makler counting chamber (Sefi Medical Instruments, Haifa, Israel), and 10 microscopic fields were subjected to each analysis in order to ensure the capture of at least 300 cells [41].

The membrane integrity of spermatozoa in the control and experimental groups was quantified by a double fluorescent staining employing CFDA (carboxyfluorescein diacetate; Sigma-Aldrich, St. Louis, MO, USA) and DAPI (4′,6-diamidino-2-phenylindole; Sigma-Aldrich, St. Louis, USA), which makes it possible to distinguish membrane intact sperm emitting green fluorescence due to a positive CFDA staining. To assess the membrane integrity, 10^6^ cells were stained with 10 μL CFDA (0.75 mg/mL in DMSO) and 10 μL DAPI (4′,6-diamidino-2-phenylindole; Sigma-Aldrich, St. Louis, MO, USA; 1 μmol/L in DPBS), and incubated at 37 °C in the dark for 15 min. Afterwards, each sample was centrifuged (150× *g*, 5 min, 20 °C) and washed with 100 µL DPBS twice. Finally, the samples were resuspended in 100 µL DPBS, transferred to 96-well microplate and subsequently measured by the combined spectro-fluoro-luminometer Glomax Multi^+^ (Promega, Madison, WI, USA) at an excitation of 490 nm and emission of 510–570 nm [17].

Acrosome integrity was analyzed by Peanut agglutinin (PNA). The principle of this method lies in a specific binding of lectin to acrosome but only in the case where acrosome is damaged. Unless the acrosome integrity is impaired, the membrane of acrosome is impermeable to lectins [79]. For the acrosome integrity, 10^6^ cells were stained with 100 μL PNA (FITC conjugate; Sigma-Aldrich, St. Louis, MO, USA; 10 μmol/L in DPBS) and 10 μL DAPI. Following incubation at 37 °C for 15 min, the samples were transferred to a 96-well plate and subjected to analysis using the Glomax Multi^+^ combined spectro-fluoro-luminometer at 490 nm excitation and 510–570 nm emission [17].

The lipophilic cation probe JC-1 (5.50, 6.60-tetrachloro-1,10,3,30 -tetraethyl benzimid azolyl carbocyanine iodide; Cayman Chemical, Ann Arbor, MI, USA) was used to assess the sperm mitochondrial membrane potential. The advantage of JC-1 lies in its capability to enter mitochondria and change its fluorescent properties. When the mitochondrial membrane is functional, JC-1 forms polymers which then emit red fluorescence. Conversely, if the membrane has a low mitochondrial potential, the dye does not form complexes and remains in its monomeric form. This is characterized by emitting green fluorescence. JC-1 dye (5.5′,6.6′-tetrachloro-1,1′,3,3′-tetraethylbenzimidazolylcarbocyanine iodide) was dissolved in DPBS creating the working solution (5 μmol/L), 5 μL of which was mixed with 10^6^ cells adjusted to 100 μL and incubated at 37 °C for 30 min. Subsequently, each sample was centrifuged (150× *g*, 5 min, 20 °C) and washed twice with DPBS. Finally, the specimens were transferred to a dark 96-well plate and analyzed using the Glomax Multi^+^ combined spectro-fluoro-luminometer [17].

Sperm DNA fragmentation was assessed using the Halomax commercial kit (Halotech DNA, Madrid, Spain). Tubes containing low-melting point agarose were placed in a water bath at 100 °C for 5 min to fuse the agarose and subsequently transferred to an incubator at 37 °C. After 5 min of incubation, 20 μL of each sample was added to the agarose. Ten microliters of the mixture were pipetted onto slides pre-coated with agarose and covered with 20 × 20 mm coverslips. The slides were then placed at 4 °C for 5 min to allow the agarose to turn into a microgel. The coverslips were gently removed, and the slides were immersed horizontally into a lysis solution (5 min). Following washing in distilled water (5 min), the slides were dehydrated in 70% and 100% ethanol (2 min each) and air-dried. All slides were stained using SYBR Green (2 μg/mL) (Sigma-Aldrich, St. Louis, USA) in Vectashield (Vector Laboratories, Burlingame, USA), and a minimum of 300 spermatozoa per sample was scored using an epifluorescence microscope with a ×40 magnification objective (Leica Microsystems, Wetzlar, Germany) [17].

### 4.3. Capacitation Patterns

Chlortetracycline (CTC) staining was used to determine the capacitation status of spermatozoa. For the assay, 784 μL of sperm suspensions adjusted to 30 × 10^6^ sperm/mL were stained with 45 μL of the CTC staining solution (750 mM CTC, 5 mM DL-cysteine, 130 mM NaCl and 20 mM Tris–HCl at pH 7.8 prepared immediately before use; Sigma-Aldrich, St. Louis, USA). The next step consisted in the addition of 8 μL 12.5% (*w*/*v*) paraformaldehyde in 0.5 M Tris–HCl (pH 7.4; Sigma-Aldrich, St. Louis, USA). Finally, 5 μL of the fixed sperm suspension was placed on a glass slide and mixed well with an equal amount of Vectashield. The droplet was then covered with a 22 × 22 mm coverslip and the slide firmly but gently pressed under two folds of a tissue paper to absorb any excess fluid. Colorless nail polish was used to seal the edges.

Evaluation of the CTC patterns was done using an epifluorescence microscope (Nikon Eclipse 80i, Tokyo, Japan) under a blue-violet illumination (excitation 330 to 380 nm, emission 420 nm). Two-hundred cells were classified into three patterns as follows: “F” pattern (intact): fluorescence detected over the entire region of the sperm head; “B” pattern (capacitated): fluorescence detected in the sperm head, except in the post-acrosomal region; and, finally, “AR” pattern (acrosome-reacted): weak fluorescence observed over the sperm head plus a bright band in the equatorial segment [14,17].

### 4.4. Oxidative Profile

Global ROS levels in the samples were assessed by the chemiluminescence assay using luminol (5-amino-2,3- dihydro-1,4-phthalazinedione; Sigma-Aldrich, St. Louis, USA) as the probe. Test samples consisted of luminol (10 μL, 5 mM) and 400 μL of control or experimental sample. The negative control was prepared by replacing the sperm suspension with 400 μL of DPBS. The positive control included 400 μL DPBS and 50 μL H_2_O_2_ (30%; 8.8 M; Sigma-Aldrich, St. Louis, USA) in triplicates. Chemiluminescence was measured on a 48-well plate for 15 min using the Glomax Multi^+^ combined spectro-fluoro-luminometer.

The nitroblue-tetrazolium (NBT) test was used to assess the intracellular formation of superoxide. Nitroblue tetrazolium chloride (2,2′-bis(4-nitrophenyl)-5,5′-diphenyl-3,3′-(3,3′-dimethoxy-4,4′diphenylene) ditetrazolium chloride; Sigma-Aldrich, St. Louis, USA) was dissolved in DPBS containing 1.5% DMSO and added to the cell suspension. After 1 h of incubation (37 °C), the cells were washed twice with DPBS and centrifuged at 300× *g* for 10 min. Finally, the suspensions were dissolved in 2 M potassium hydroxide (KOH; Centralchem, Bratislava, Slovakia) in DMSO. The optical density was determined at a wavelength of 620 nm against 570 nm as a reference using the Multiskan FC microplate photometer (Thermo Fisher Scientific; Waltham, MA, USA). The collected data were expressed in percentage of the control, which was set to 100%.

The Amplex^®^ Red reagent (10-acetyl-3,7-dihydroxyphenoxazine; Thermo Fisher Scientific; Waltham, MA, USA) in combination with horseradish peroxidase (HRP; Thermo Fisher Scientific; Waltham, MA, USA) was used to detect H_2_O_2_ released from spermatozoa in the control and experimental groups. In the presence of peroxidase, the reagent reacts with H_2_O_2_ in a 1:1 stoichiometry to produce the red fluorescent resorufin. Resorufin has excitation and emission maxima of approximately 571 nm and 585 nm. A working solution consisting of 10 mM Amplex Red^®^ and 10 UI HRP was used to treat 10^6^ cells adjusted to 50 μL, and the mixture was incubated at 37 °C for 30 min. The amount of resofurin was detected with the Glomax Multi^+^ combined spectro-fluoro-luminometer [17].

Aminophenyl fluorescein (APF; Thermo Fisher Scientific; Waltham, MA, USA) was used to quantify the production of the hydroxyl radical. Upon oxidation, APF emits bright green fluorescence (excitation/ emission maxima ~490/515 nm), making it compatible with fluorescence instrumentation capable of visualizing fluorescein. One million cells adjusted to 100 μL were stained with 2 μL of 100 μM APF staining solution and incubated at 37 °C for 20 min. The intensity of APF fluorescence was analyzed with the Glomax Multi^+^ combined spectro-fluoro-luminometer [17].

### 4.5. Western Blots

For the extraction of sperm proteins, the cell suspensions were processed with a single-layer Percoll^®^ Plus (Sigma-Aldrich, St. Louis, MO, USA) density gradient separation, according to Ďuračka et al. [80]. Subsequently, the cells were treated with 1 mL RIPA buffer (Sigma-Aldrich, St. Louis, MO, USA) with protease inhibitor (Sigma-Aldrich, St. Louis, MO, USA), thoroughly mixed with the buffer and left in the refrigerator overnight. The next day, the samples were mixed again and centrifuged at 11,828× *g* for 10 min at 4 °C. Collected supernatants were subjected to the determination of proteins, based on the Biuret reaction. A commercial total protein kit (DiaSys, Holzheim, Germany) was used, and the assay was performed with the RX Monza photometer (Randox, Crumlin, UK) [15].

Protein concentration in the lysates was adjusted using ultrapure (UHQ) water to reach a final concentration of 25 μg protein/20 μL sample. The samples were treated with 4× Laemli buffer (BioRad, Hercules, CA, USA) and β-mercaptoethanol (Sigma-Aldrich, St. Louis, MO, USA) and then boiled at 95 °C for 10 min. Subsequently, the samples were loaded (20 μL) into Mini-PROTEAN TGX stain-free polyacrylamide gels (BioRad, Hercules, CA, USA), along with 7 μL of Precision Plus Protein marker (BioRad, Hercules, CA, USA). The electrophoresis was run at 90 V for 2 h, and the gels were visualized with the ChemiDoc Imaging System (BioRad, Hercules, CA, USA) to confirm the loading uniformity. For the blotting procedure, the gels were transferred to PVDF membranes (Trans-Blot Turbo Pack; BioRad, Hercules, CA, USA) using the Trans-Blot Turbo Transfer System (BioRad, Hercules, CA, USA), 25 V, 2.5 A, for 7 min (for CatSper1/2 and PKA) or 10 min (for NBC). After completion of the blot, the membranes were incubated for 3 × 10 min in Tris-buffered saline (TBS), composed of Tris base (Sigma-Aldrich, St. Louis, MO, USA), sodium chloride (Centralchem, Bratislava, Slovakia) and UHQ water. This step was followed by membrane staining with Ponceau S solution (Sigma-Aldrich, St. Louis, MO, USA) to visualize the bands on the membranes. Subsequently, the membranes were cut into smaller pieces, where the protein of interest was presumably localized. The membrane pieces were then de-stained with TBS (3 × 10 min) and blocked with 5% skim milk (Blotting grade blocker; BioRad, Hercules, CA, USA) in TBS containing 0.1% Tween-20 (Sigma-Aldrich, St. Louis, MO, USA). Membrane blocking was performed on a stirrer at room temperature for 2 h. Finally, the membranes were incubated with one of the primary antibodies described in Table 1.

The next day, the membranes were washed for 5 × 10 min in wash buffer composed of 1% milk in TBS/0.2% Tween-20, and subsequently incubated with a secondary anti-rabbit antibody (GE Healthcare, Chicago, IL, USA) diluted 1:15,000 in 1% milk in TBS/0.2% Tween-20 for 1 h. Following incubation, the membranes were washed for 3 × 10 min in TBS/0.2% Tween-20 at room temperature and using a stirrer. To visualize the protein bands, the membranes were incubated with the ECL substrate (GE Healthcare, Chicago, IL, USA) in the dark for 5 min. After incubation, the membranes were placed into the ChemiDoc Imaging System, which automatically calculated the protein visualization time based on the light signal emitted by the membranes [14]. Protein expression was evaluated using BioRad Image Lab Software 6.1 (BioRad, Hercules, CA, USA).

### 4.6. Statistics

Statistical analysis was carried out using the GraphPad Prism program (version 9.2.0 for Mac; GraphPad Software, La Jolla, CA, USA). One-way ANOVA was used for statistical evaluations. Dunnett’s test was selected as a follow-up test to ANOVA, based on a comparison of every mean to a control mean, and creating a confidence interval for the difference between the two means. The level of significance was set at **** *p* < 0.0001, *** *p* < 0.001, ** *p* < 0.01 and * *p* < 0.05. The comparative analysis was performed as follows:

- Native control (CtrlN) was compared to the cryopreserved control (CtrlC);
- Experimental groups were compared to both controls.

## 5. Conclusions

In conclusion, we may provide support to earlier studies suggesting that the molecular machinery of cryocapacitation is primarily driven by membrane disintegration due to thermal shock, ice crystals and oxidative stress, which will cause inactivation of the transmembrane channels and a subsequent decrease of the enzymatic activity responsible for sperm hyperactivation. At the same time, epicatechin seems to exert its antioxidant activity primarily in the sperm membrane, disposing of ROS involved in lipid peroxidation, thus stabilizing the structural and molecular machinery responsible for a proper capacitation process to occur when needed. Adequate protection of the structures and molecules crucial for the sperm structural integrity and functional activity provided by epicatechin will then lead to a higher proportion of spermatozoa with a desirable post-thaw quality.

## Figures and Tables

**Table 1:** Antibodies used in the Western blot analysis.

Target Protein	Antibody	Source	Clonality/Isotype	Dilution	Blocking Solution	Cat. #	Manufacturer
CatSper1	CATSPER1 Polyclonal Antibody	rabbit	Polyclonal/IgG	1:1000	5% milk/TBS/0.1% Tween-20	PA5-75788	Invitrogen, Waltham, MA, USA
CatSper2	CATSPER2 Polyclonal Antibody	rabbit	Polyclonal/IgG	1:1000	5% milk/TBS/0.1% Tween-20	PA5-41072	Invitrogen, Waltham, MA, USA
NBC	Anti-Na^+^/HCO_3_^−^ Contransporter Polyclonal Antibody	rabbit	Polyclonal/IgG	1:500	5% milk/TBS/0.1% Tween-20	AB3212-I	Merck Millipore, Temecula, CA, USA
PKA	PKA alpha Antibody	rabbit	Polyclonal/IgG	1:1000	5% milk/TBS/0.1% Tween-20	PA5-17626	Invitrogen, Waltham, MA, USA
β-actin	beta Actin Polyclonal Antibody	rabbit	Polyclonal/IgG	1:1000	5% milk/TBS/0.1% Tween-20	PA1-46296	Invitrogen, Waltham, MA, USA

## Data Availability

The data presented in this study are available upon reasonable request from the corresponding author.

## Supplementary Materials

The following are available online at https://www.mdpi.com/article/10.3390/ijms24032510/s1.

## References

[1] Hezavehei M., Sharafi M., Kouchesfahani H.M., Henkel R., Agarwal A., Esmaeili V., Shahverdi A. (2018). Sperm cryopreservation: A review on current molecular cryobiology and advanced approaches. Reprod. Biomed. Online.

[2] Curry M.R. (2000). Cryopreservation of semen from domestic livestock. Rev. Reprod..

[3] Peris-Frau P., Soler A.J., Iniesta-Cuerda M., Martín-Maestro A., Sánchez-Ajofrín I., Medina-Chávez D.A., Fernández-Santos M.R., García-Álvarez O., Maroto-Morales A., Montoro V. (2020). Sperm Cryodamage in Ruminants: Understanding the Molecular Changes Induced by the Cryopreservation Process to Optimize Sperm Quality. Int. J. Mol. Sci..

[4] Ombelet W., Van Robays J. (2015). Artificial insemination history: Hurdles and milestones. Facts Views Vis. Obgyn..

[5] John Morris G., Acton E., Murray B.J., Fonseca F. (2012). Freezing injury: The special case of the sperm cell. Cryobiology.

[6] Ambar R.F., Gava M.M., Ghirelli-Filho M., Yoshida I.H., De Paula T.S., Glina S. (2021). Tissue and sperm handling before assisted reproductive technology (ART): A systematic review. Arab J. Urol..

[7] Vigolo V., Giaretta E., Da Dalt L., Damiani J., Gabai G., Bertuzzo F., Falomo M.E. (2022). Relationships between Biomarkers of Oxidative Stress in Seminal Plasma and Sperm Motility in Bulls before and after Cryopreservation. Animals.

[8] Aitken R.J., Bromfield E.G., Gibb Z. (2022). Oxidative stress and reproductive function: The impact of oxidative stress on reproduction: A focus on gametogenesis and fertilization. Reproduction.

[9] Arjun V., Kumar P., Dutt R., Kumar A., Bala R., Verma N., Jerome A., Virmani M., Patil C.S., Singh S. (2021). Is addition or removal of seminal plasma able to compensate for the dilution effect of buffalo semen?. Andrologia.

[10] Castiglioni V.C., Siqueira A.F.P., Bicudo L.C., de Almeida T.G., Hamilton T.R.D.S., de Castro L.S., Mendes C.M., Nichi M., Losano J.D.A., Visitin J.A. (2021). Lipid peroxidation in bull semen influences sperm traits and oxidative potential of Percoll^®^-selected sperm. Zygote.

[11] Tvrda E., Straka P., Galbavy D., Ivanic P. (2019). Epicatechin Provides Antioxidant Protection to Bovine Spermatozoa Subjected to Induced Oxidative Stress. Molecules.

[12] Ribas-Maynou J., Delgado-Bermúdez A., Mateo-Otero Y., Viñolas E., Hidalgo C.O., Ward W.S., Yeste M. (2022). Determination of double- and single-stranded DNA breaks in bovine sperm is predictive of their fertilizing capacity. J. Anim. Sci. Biotechnol..

[13] Arif A.A., Maulana E.M., Kaiin E.M., Purwantara B., Arifiantini R.I. (2022). The quality of frozen semen of Limousin bull in various semen diluetnts. Trop. Anim. Sci. J..

[14] Benko F., Fialková V., Žiarovská J., Ďuračka M., Lukáč N., Tvrdá E. (2022). In Vitro versus Cryo-Induced Capacitation of Bovine Spermatozoa, Part 2: Changes in the Expression Patterns of Selected Transmembrane Channels and Protein Kinase A. Int. J. Mol. Sci..

[15] Visconti P.E. (2009). Understanding the molecular basis of sperm capacitation through kinase design. Proc. Natl. Acad. Sci. USA.

[16] Rajoriya J.S., Prasad J.K., Ramteke S.S., Perumal P., De A.K., Ghosh S.K., Bag S., Raje A., Singh M., Kumar A. (2020). Exogenous cholesterol prevents cryocapacitation-like changes, membrane fluidity, and enhances in vitro fertility in bubaline spermatozoa. Reprod. Domest. Anim..

[17] Benko F., Mohammadi-Sangcheshmeh A., Ďuračka M., Lukáč N., Tvrdá E. (2022). In vitro versus cryo-induced capacitation of bovine spermatozoa, part 1: Structural, functional, and oxidative similarities and differences. PLoS ONE.

[18] Sieme H., Oldenhof H., Wolkers W.F. (2016). Mode of action of cryoprotectants for sperm preservation. Anim. Reprod. Sci..

[19] Bahmyari R., Zare M., Sharma R., Agarwal A., Halvaei I. (2020). The efficacy of antioxidants in sperm parameters and production of reactive oxygen species levels during the freeze-thaw process: A systematic review and meta-analysis. Andrologia.

[20] Robles V., Valcarce D.G., Riesco M.F. (2019). The Use of Antifreeze Proteins in the Cryopreservation of Gametes and Embryos. Biomolecules.

[21] Mandal R., Badyakar D., Chakrabarty J. (2014). Role of Membrane Lipid Fatty Acids in Sperm Cryopreservation. Adv. Androl..

[22] Barakat I.A., Danfour M.A., Galewan F.A., Dkhil M.A. (2015). Effect of various concentrations of caffeine, pentoxifylline, and kallikrein on hyperactivation of frozen bovine semen. BioMed Res. Int..

[23] Boroujeni S.N., Malamiri F.A., Bossaghzadeh F., Esmaeili A., Moudi E. (2022). The most important medicinal plants affecting sperm and testosterone production: A systematic review. JBRA Assist. Reprod..

[24] Roozbeh N., Amirian A., Abdi F., Haghdoost S. (2021). A Systematic Review on Use of Medicinal Plants for Male Infertility Treatment. J. Family Reprod. Health.

[25] Tvrdá E., Benko F., Slanina T., du Plessis S.S. (2021). The Role of Selected Natural Biomolecules in Sperm Production and Functionality. Molecules.

[26] Dos Santos A.N., de Nascimento T.R.L., Gondim B.L.C., Velo M.M.A.C., de Rêgo R.I.A., do Neto J.R.C., Machado J.R., da Silva M.V., de Araújo H.W.C., Fonseca M.G. (2020). Catechins as Model Bioactive Compounds for Biomedical Applications. Curr. Pharm. Des..

[27] Roychoudhury S., Agarwal A., Virk G., Cho C.L. (2017). Potential role of green tea catechins in the management of oxidative stress-associated infertility. Reprod. Biomed. Online.

[28] Isemura M. (2019). Catechin in Human Health and Disease. Molecules.

[29] Pirker K.F., Baratto M.C., Basosi R., Goodman B.A. (2012). Influence of pH on the speciation of copper (II) in reactions with the green tea polyphenols, epigallocatechin gallate and gallic acid. J. Inorg. Biochem..

[30] Zwolak I. (2021). Epigallocatechin Gallate for Management of Heavy Metal-Induced Oxidative Stress: Mechanisms of Action, Efficacy, and Concerns. Int. J. Mol. Sci..

[31] Rai A., Gill M., Kinra M., Dsouza L.A., Sumalatha S., Raj S., Shetty R., Nandakumar K., Chamallamudi M.R., Kumar N. (2020). Assessment of preclinical effect of (+)-catechin hydrate on sexual function: An in silico and in vivo study. Andrologia.

[32] Ding J., Wang H., Wu Z.-B., Zhao J., Zhang S., Li W. (2015). Protection of murine spermatogenesis against ionizing radiation-induced testicular injury by a green tea polyphenol. Biol. Reprod..

[33] Abshenas J., Babaei H., Zare M.H., Allahbakhshi A., Sharififar F. (2012). The effects of green tea (*Camellia sinensis*) extract on mouse semen quality after scrotal heat stress. Vet. Res. Forum.

[34] Zanchi M.M., Manfredini V., Dos Santos Brum D., Vargas L.M., Spiazzi C.C., Soares M.B., Izaguirry A.P., Santos F.W. (2015). Green tea infusion improves cyclophosphamide-induced damage on male mice reproductive system. Toxicol. Rep..

[35] Awoniyi D.O., Aboua Y.G., Marnewick J., Brooks N. (2012). The effects of rooibos (*Aspalathus linearis*), green tea (*Camellia sinensis*) and commercial rooibos and green tea supplements on epididymal sperm in oxidative stress-induced rats. Phytother. Res..

[36] Silva E.C.B., Arruda L.C.P., Vieira J.I.T., Soares P.C., Guerra M.M.P. (2019). (+)-Catechin and (-)-epigallocatechin gallate: Are these promising antioxidant therapies for frozen goat semen?. Arq. Bras. Med. Vet. Zootec..

[37] Boonsorn T., Kongbuntad W., Narkkong N., Aengwanich W. (2010). Effects of catechin addition to extender on sperm quality and lipid peroxidation in boar semen. Am. Eurasian J. Sustain. Agric..

[38] Wittayarat M., Ito A., Kimura T., Namula Z., Luu V.V., Do L.T., Sato Y., Taniguchi M., Otoi T. (2013). Effects of green tea polyphenol on the quality of canine semen after long-term storage at 5 °C. Reprod. Biol..

[39] Moretti E., Mazzi L., Terzuoli G., Bonechi C., Iacoponi F., Martini S., Rossi C., Collodel G. (2012). Effect of quercetin, rutin, naringenin and epicatechin on lipid peroxidation induced in human sperm. Reprod. Toxicol..

[40] Jamalan M., Ghaffari M.A., Hoseinzadeh P., Hashemitabar M., Zeinali M. (2016). Human Sperm Quality and Metal Toxicants: Protective Effects of some Flavonoids on Male Reproductive Function. Int. J. Fertil. Steril..

[41] Baňas Š., Ďuračka M., Benko F., Žiarovská J., Lukáč N., Tvrdá E. (2022). Epicatechin improves frozen sperm vitality by its antioxidant and cryoprotective actions. J. Microbiol. Biotech. Food Sci..

[42] De Amicis F., Santoro M., Guido C., Russo A., Aquila S. (2012). Epigallocatechin gallate affects survival and metabolism of human sperm. Mol. Nutr. Food Res..

[43] Ugur M.R., Saber Abdelrahman A., Evans H.C., Gilmore A.A., Hitit M., Arifiantini R.I., Purwantara B., Kaya A., Memili E. (2019). Advances in Cryopreservation of Bull Sperm. Front. Vet. Sci..

[44] De Leeuw F.E., Chen H.C., Colenbrander B., Verkleij A.J. (1990). Cold-induced ultrastructural changes in bull and boar sperm plasma membranes. Cryobiology.

[45] Khalil W.A., El-Harairy M.A., Zeidan A.E.B., Hassan M.A.E., Mohey-Elsaeed O. (2017). Evaluation of bull spermatozoa during and after cryopreservation: Structural and ultrastructural insights. Int. J. Vet. Sci. Med..

[46] Ahmad M., Ahmad N., Riaz A., Anzar M. (2015). Sperm survival kinetics in different types of bull semen: Progressive motility, plasma membrane integrity, acrosomal status and reactive oxygen species generation. Reprod. Fertil. Dev..

[47] Nagata M.B., Egashira J., Katafuchi N., Endo K., Ogata K., Yamanaka K., Yamanouchi T., Matsuda H., Hashiyada Y., Yamashita K. (2019). Bovine sperm selection procedure prior to cryopreservation for improvement of post-thawed semen quality and fertility. J. Anim. Sci. Biotechnol..

[48] Madeja Z.E., Podralska M., Nadel A., Pszczola M., Pawlak P., Rozwadowska N. (2021). Mitochondria Content and Activity Are Crucial Parameters for Bull Sperm Quality Evaluation. Antioxidants.

[49] Trevizan J.T., Carreira J.T., Carvalho I.R., Kipper B.H., Nagata W.B., Perri S.H.V., Franco Oliveira M.E., Pierucci J.C., Koivisto M.B. (2018). Does lipid peroxidation and oxidative DNA damage differ in cryopreserved semen samples from young, adult and aged Nellore bulls?. Anim. Reprod. Sci..

[50] Benko F., Duracka M., Lukac N., Tvrda E. (2022). Cryocapacitation and its association with oxidative features in cryopreserved bovine spermatozoa. J. Microbiol. Biotech. Food Sci..

[51] Martin G., Sabido O., Durand P., Levy R. (2004). Cryopreservation induces an apoptosis-like mechanism in bull sperm. Biol. Reprod..

[52] Alshawa E., Laqqan M., Montenarh M., Hammadeh M.E. (2019). Influence of cryopreservation on the CATSPER2 and TEKT2 expression levels and protein levels in human spermatozoa. Toxicol. Rep..

[53] Jensen L.J., Schmitt B.M., Berger U.V., Nsumu N.N., Boron W.F., Hediger M.A., Brown D., Breton S. (1999). Localization of sodium bicarbonate cotransporter (NBC) protein and messenger ribonucleic acid in rat epididymis. Biol. Reprod..

[54] Flores E., Ramió-Lluch L., Bucci D., Fernández-Novell J.M., Peña A., Rodríguez-Gil J.E. (2011). Freezing-thawing induces alterations in histone H1-DNA binding and the breaking of protein-DNA disulfide bonds in boar sperm. Theriogenology.

[55] Harrison R.A., White I.G. (1972). Glycolytic enzymes in the spermatozoa and cytoplasmic droplets of bull, boar and ram, and their leakage after shock. J. Reprod. Fertil..

[56] Lee-Estevez M., Herrera L., Díaz R., Beltrán J., Figueroa E., Dumorné K., Ulloa-Rodríguez P., Short S., Risopatrón J., Valdebenito I. (2019). Effects of cryopreservation on cAMP-dependent protein kinase and AMP-activated protein kinase in Atlantic salmon (Salmo salar) spermatozoa: Relation with post-thaw motility. Anim. Reprod. Sci..

[57] Feliciello A., Gottesman M.E., Avvedimento E.V. (2001). The biological functions of A-kinase anchor proteins. J. Mol. Biol..

[58] Stival C., Ritagliati C., Xu X., Gervasi M.G., Luque G.M., Baró Graf C., De la Vega-Beltrán J.L., Torres N., Darszon A., Krapf D. (2018). Disruption of protein kinase A localization induces acrosomal exocytosis in capacitated mouse sperm. J. Biol. Chem..

[59] Ahmadi S., Bashiri R., Ghadiri-Anari A., Nadjarzadeh A. (2016). Antioxidant supplements and semen parameters: An evidence-based review. Int. J. Reprod. Biomed..

[60] Silvestre M.A., Yániz J.L., Peña F.J., Santolaria P., Castelló-Ruiz M. (2021). Role of Antioxidants in Cooled Liquid Storage of Mammal Spermatozoa. Antioxidants.

[61] Al-Mutary M.G. (2021). Use of antioxidants to augment semen efficiency during liquid storage and cryopreservation in livestock animals: A review. J. King Saud Univ. Sci..

[62] Zhang Y., Lin H., Liu C., Huang J., Liu Z. (2020). A review for physiological activities of EGCG and the role in improving fertility in humans/mammals. Biomed. Pharmacother..

[63] Kim H.S., Quon M.J., Kim J.A. (2014). New insights into the mechanisms of polyphenols beyond antioxidant properties; lessons from the green tea polyphenol, epigallocatechin 3-gallate. Redox. Biol..

[64] Nagle D.G., Ferreira D., Zhou Y.D. (2006). Epigallocatechin-3-gallate (EGCG): Chemical and biomedical perspectives. Phytochemistry.

[65] Galleano M., Verstraeten S.V., Oteiza P.I., Fraga C.G. (2010). Antioxidant actions of flavonoids: Thermodynamic and kinetic analysis. Arch. Biochem. Biophys..

[66] Uekusa Y., Kamihira M., Nakayama T. (2007). Dynamic behavior of tea catechins interacting with lipid membranes as determined by NMR spectroscopy. J. Agric. Food Chem..

[67] Greifova H., Tvrda E., Jambor T., Lukac N. (2018). Dose- and time-dependent effects of epicatechin on bovine spermatozoa in vitro. J. Microbiol. Biotech. Food Sci..

[68] Purdy P.H., Ericsson S.A., Dodson R.E., Sternes K.L., Garner D.L. (2004). Effects of flavonoids, silibinin and catechin, on the motility of extended cooled caprine sperm. Small Rumin. Res..

[69] Heo S., Kim S., Kang D. (2020). The Role of Hydrogen Peroxide and Peroxiredoxins throughout the Cell Cycle. Antioxidants.

[70] Tejero I., Gonzalez-Lafont A., Lluch J.M., Eriksson L.A. (2007). Theoretical modeling of hydroxyl-radical-induced lipid peroxidation reactions. J. Phys. Chem. B..

[71] Zarkovic N. (2018). Antioxidants and Second Messengers of Free Radicals. Antioxidants.

[72] Gough D., Cotter T. (2011). Hydrogen peroxide: A Jekyll and Hyde signalling molecule. Cell Death. Dis..

[73] Mandel S.A., Amit T., Kalfon L., Reznichenko L., Weinreb O., Youdim M.B. (2008). Cell signaling pathways and iron chelation in the neurorestorative activity of green tea polyphenols: Special reference to epigallocatechin gallate (EGCG). J. Alzheimers Dis..

[74] Sapanidou V.G., Margaritis I., Siahos N., Arsenopoulos K., Dragatidou E., Taitzoglou I.A., Zervos I.A., Theodoridis A., Tsantarliotou M.P. (2014). Antioxidant effect of a polyphenol-rich grape pomace extract on motility, viability and lipid peroxidation of thawed bovine spermatozoa. J. Biol. Res..

[75] Gale I., Gil L., Malo C., González N., Martínez F. (2015). Effect of Camellia sinensis supplementation and increasing holding time on quality of cryopreserved boar semen. Andrologia.

[76] Naaby-Hansen S., Herr J.C. (2010). Heat shock proteins on the human sperm surface. J. Reprod. Immunol..

[77] Degenhardt K., Sundararajan R., Lindsten T., Thompson C., White E. (2002). Bax and Bak independently promote cytochrome C release from mitochondria. J. Biol. Chem..

[78] Spinaci M., Volpe S., De Ambrogi M., Tamanini C., Galeati G. (2008). Effects of epigallocatechin-3-gallate (EGCG) on in vitro maturation and fertilization of porcine oocytes. Theriogenology.

[79] Paál D., Strejček F., Tvrdá E., Formicki G., Klein S., Rath D., Massanyi P. (2018). The in Vitro Effect of Taurine on Boar Spermatozoa Quality. Acta Univ. Agric. Silvic. Mendelianae Brun..

[80] Ďuračka M., Husarčíková K., Jančov M., Galovičová L., Kačániová M., Lukáč N., Tvrdá E. (2021). Staphylococcus-Induced Bacteriospermia In Vitro: Consequences on the Bovine Spermatozoa Quality, Extracellular Calcium and Magnesium Content. Animals.

